# Reactivation of corticogenesis-related transcriptional factors BCL11B and SATB2 after ischemic lesion of the adult mouse brain

**DOI:** 10.1038/s41598-023-35515-8

**Published:** 2023-05-26

**Authors:** Sanja Srakočić, Dunja Gorup, Dominik Kutlić, Ante Petrović, Victor Tarabykin, Srećko Gajović

**Affiliations:** 1 Croatian Institute for Brain Research, University of Zagreb School of Medicine, Šalata 12, 10000 Zagreb, Croatia; 2 Universität Zürich, Universitätspital Zürich, Zürich, Switzerland; 3 Institute of Cell Biology and Neurobiology, Charité-Universitätsmedizin, Berlin, Germany; 4 Institute of Neuroscience, University of Nizhny Novgorod, Pr. Gagarina 24, Nizhny Novgorod, Russia

**Keywords:** Regeneration and repair in the nervous system, Experimental models of disease, Stroke, Preclinical research, Stroke, Neuronal development

## Abstract

The aim of this study was to characterize expression of corticogenesis-related transcription factors BCL11B and SATB2 after brain ischemic lesion in the adult mice, and to analyze their correlation to the subsequent brain recovery. Ischemic brain lesion was induced by transient middle cerebral artery occlusion followed by reperfusion, and the animals with ischemic lesion were compared to the sham controls. Progression of the brain damage and subsequent recovery was longitudinally monitored structurally, by magnetic resonance imaging, and functionally, by neurological deficit assessment. Seven days after the ischemic injury the brains were isolated and analyzed by immunohistochemistry. The results showed higher expression in the brain of both, BCL11B and SATB2 in the animals with ischemic lesion compared to the sham controls. The co-expression of both markers, BCL11B and SATB2, increased in the ischemic brains, as well as the co-expression of BCL11B with the beneficial transcriptional factor ATF3 but not its co-expression with detrimental HDAC2. BCL11B was mainly implicated in the ipsilateral and SATB2 in the contralateral brain hemisphere, and their level in these regions correlated with the functional recovery rate. The results indicate that the reactivation of corticogenesis-related transcription factors BCL11B and SATB2 is beneficial after brain ischemic lesion.

## Introduction

In the course of corticogenesis young neurons born from their progenitors differentiate into specific neuronal cell types that project to various target areas in the CNS. In mice it occurs in the second half of gestation, between days E11 and E19. Differentiation of neuronal cell types is regulated by a set of transcriptional factors specific for each cell type^1^. Transcriptional factors BCL11B (B-cell lymphoma/leukemia 11B, aka CTIP2—chicken ovalbumin upstream promoter transcription factor interacting protein 2) and SATB2 (special AT-rich sequence binding protein 2) are key determinants of two major neuronal subclasses: cortico-subcortical (BCL11B) and cortico-cortical (SATB2). Since both interact with the chromatin remodeling complexes, they are capable of altering chromatin structure and simultaneously modify the expression of multiple genes^2,3^. BCL11B is a zinc finger transcriptional factor primarily responsible for establishing the correct connectivity of lower cortical layer neurons, mainly of layer 5 motor neurons^2^. On the other hand, SATB2 is essential for the establishment of cortico-cortical interhemispheric connections between the upper-layers neuron^4,5^. *Satb2* deficiency is postnatal lethal and causes multiple defects including impaired development of corpus callosum, the major axonal tract interconnecting two cerebral hemispheres^4^. BCL11B and SATB2 interact with each other during corticogenesis. SATB2 acts as a negative regulator of *Bcl11b* by recruiting NuRD (the nucleosome remodeling and deacetylase) histone remodeling complex on *Bcl11b* locus, which deacetylates the histones thus impairing the transcription^4,6^. However, some cells in the lower layers of neocortex express both transcriptional factors since on one hand LMO4 (LIM domain only protein 4) prevents the recruitment of SATB2 on *Bcl11b* locus, on the other hand a transcriptional co-factor Ski is required for SATB2 to repress BCL11B^7^. These double-positive neurons project either to contralateral brain hemisphere via corpus callosum or into the brain stem nuclei^8^. Besides their involvement in neocortical circuits, BCL11B and SATB2 have also been found in other brain regions, and their expression persists even in the adult brain. BCL11B is also associated with the differentiation of medium spiny neurons in striatum during embryonic development^9^. Furthermore, in adult brain BCL11B is expressed in basal ganglia, fifth cranial ganglion, olfactory bulb and in spinal cord^10^. In the adult mouse brain, SATB2 expression was reported in neurons of all layers in neocortex, as well as in various sub-cortical structures, including majority of hypothalamic nuclei and the dorsal raphe nuclei, along with the expression in neocortex^4,11^.

The possibility that corticogenesis-related transcription factors can act in the adult brain, in particular in damage repair can be relevant for the regenerative neuroscience. Brain stroke is a leading cause of death and disabilities worldwide with the increasing incidence and major economic burden^12^. Brain stroke is characterized by a rapid loss of brain circuitry and respective functions. In most cases it has vascular origin^13^. Most of strokes (70%) are ischemic, while the rest represent hemorrhagic strokes. The therapeutic options of ischemic stroke remain limited to thrombectomy, intravenous thrombolysis and patient care in the specialized stroke units^12,14^. Vast majority of potential therapeutics have been tested in animal models, however none of them proved effective in the clinical trials^15^. The challenge of neuroprotective treatment for brain ischemia is the activation of complex, interconnected network of pathophysiological processes that can have either beneficial effect or cause additional damage^16^. Oxygen deficiency impairs cellular metabolism leading to excitotoxicity and oxidative damage. As a result, cell death occurs further initiating the process of inflammation and causing breakdown of the blood–brain-barrier^16,17^. The lack of appropriate neuroprotective therapies suggests there is still a lot to be discovered how neurons can counteract these unfavorable conditions. Identification and further research of unexplored pathways associated with the repair of the brain damage caused by the ischemic stroke might open additional possibilities for the development of novel therapeutic approaches.

The aim of this study was to test if there are changes in the expression of corticogenesis-related transcriptional factors BCL11B and SATB2 after ischemic brain lesion in the adult mice. We hypothesized that their reactivation may be associated with the brain recovery after ischemia-related damage. Both BCL11B and SATB2 control the expression of large number of diverse genes and subsequently can contribute to significant change of the protein profile after brain ischemia^18^. To relate expression of BCL11B and SATB2 to the recovery processes after ischemic stroke, selecting appropriate time point for their analysis after ischemia onset was crucial. The acute phase of ischemic stroke, first few days after the onset, is defined by cell death, activation of inflammation processes and severe brain edema that contribute to the brain damage^19–22^. Subsequently brain edema diminishes, and lesion volume subsides^22,23^. The sub-acute phase is followed by a chronic phase characterized by a decrease of inflammation, consolidation of the ischemic core and an increase of brain plasticity markers along with functional recovery^20,22,24^. To investigate the expression of BCL11B and SATB2, 7 days after ischemic injury was chosen as the turning point between sub-acute and chronic phases. Moreover, we could simultaneously assess the dynamic of brain changes by using in vivo magnetic resonance imaging (MRI) to measure the lesion size and calculate its reduction rate between successive time points. The imaging results were additionally complemented with the neurological deficit assessment and the neurological recovery rate between same time points was calculated accordingly. Our results showed an increased expression of BCL11B and SATB2 after brain ischemic lesion, which correlated with the functional recovery rate in the analyzed animals.

## Results

The study design included two experimental groups, MCAO (n = 6) and Sham (n = 4) group. MCAO group had ischemic lesion induced by tMCAO (transient Middle Cerebral Artery occlusion), while Sham group was operated in the same way but without inducing ischemia. Both experimental groups underwent brain in vivo MRI, and neurological deficit tests on days 3 and 7 after MCAO. The animals were sacrificed 7 days after MCAO, and their brains were analyzed by immunohistochemistry, which was subsequently correlated to the volumes of the ischemic lesion and the corresponding neurological scores.

### Increased expression of BCL11B and SATB2 after ischemic brain lesion in mice

To study the BCL11B and SATB2 expression in the adult mouse brain after ischemic lesion, MCAO animals were compared to Sham controls 7 days after brain ischemia. Immunohistochemistry revealed a spatial pattern of BCL11B and SATB2 expression after brain ischemia and the results were quantified by measuring integrated optical density of the labelled cells. Integrated optical density was measured individually in the regions of neocortex, striatum, and hippocampus surrounding the necrotic ischemic core, and in the same regions of the contralateral brain hemisphere. Subsequently, the obtained data from different brain regions were combined to analyze the total expression for brain hemispheres and for the entire brain.

**Figure 1:**
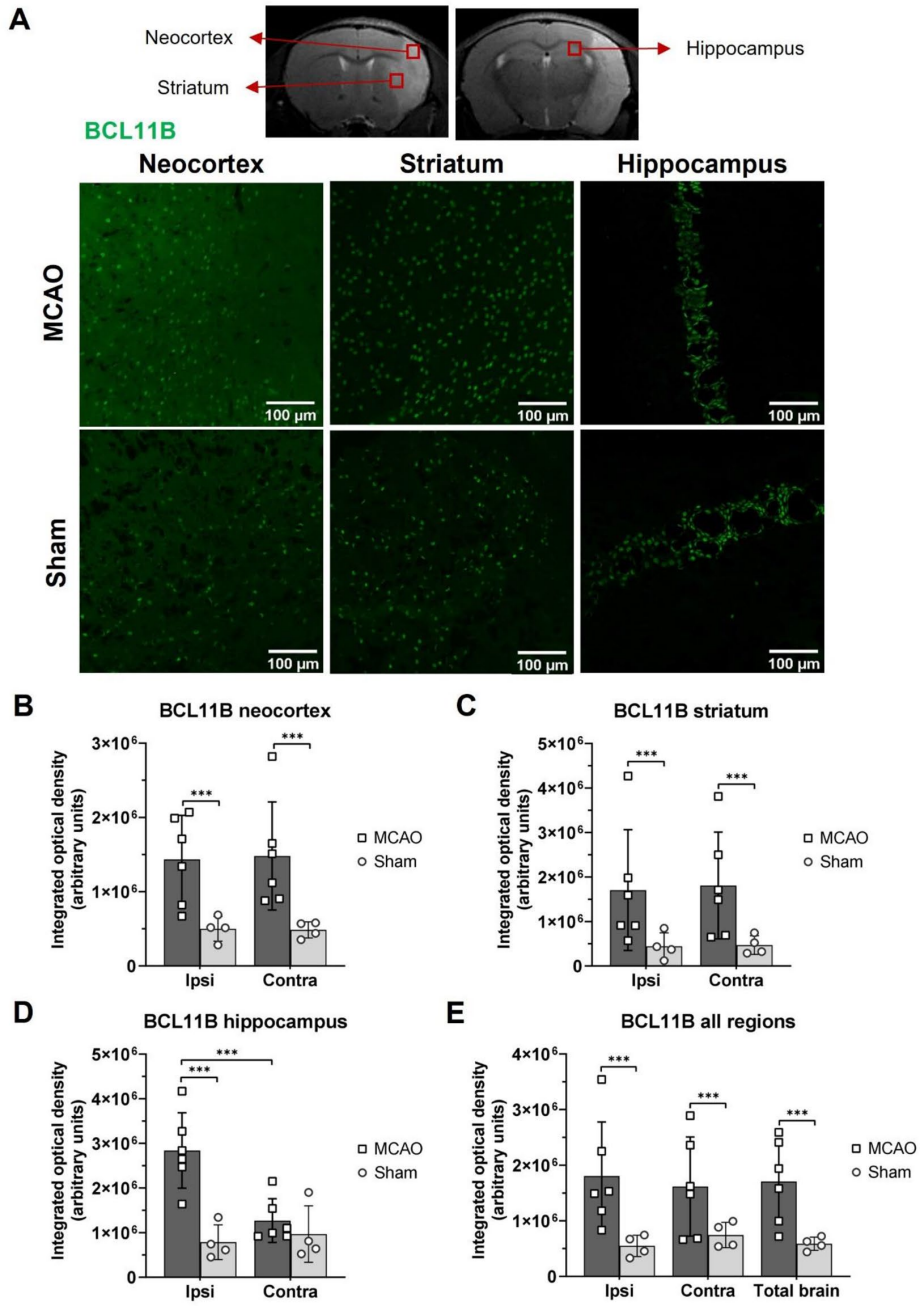
Immunohistochemistry results of BCL11B in the mouse brains isolated 7 days after MCAO or Sham surgery. (**A**) Representative immunofluorescent images of BCL11B (green) expression in neocortex, striatum, and hippocampus of MCAO and Sham experimental groups. The images were taken from the ipsilateral brain hemisphere. Regions of interest displayed on immunofluorescent images are marked with the red square on the representative MR image of the mouse brain. (**B–D**) Results of the integrated optical density measurements of BCL11B fluorescent signal in neocortex (**B**), striatum (**C**) and hippocampus (**D**) of MCAO and Sham experimental groups. Expression of BCL11B was higher in MCAO operated animals, in all of the analyzed regions, except contralateral hippocampus. (**E**) Integrated optical density of BCL11B calculated for whole brain hemispheres and entire brain of MCAO and Sham experimental groups showing higher levels of BCL11B. The statistical significance was tested using nested ANOVA followed by Tukey’s multiple comparisons correction. Data are presented as mean ± standard deviation. ***P < 0.001.

BCL11B signal was observed in both experimental groups, MCAO and Sham. BCL11B was higher in the MCAO group than in Sham group in all examined regions of the ipsilateral hemisphere and in the neocortex and striatum of the contralateral hemisphere (Fig. 1, Supplemental Table 1). The combined data showed higher BCL11B levels in both brain hemispheres accordingly, and in the entire brain than in Sham animals. Taken together, these results indicated an increase in BCL11B brain expression as a result of ischemic brain injury.

**Figure 2:**
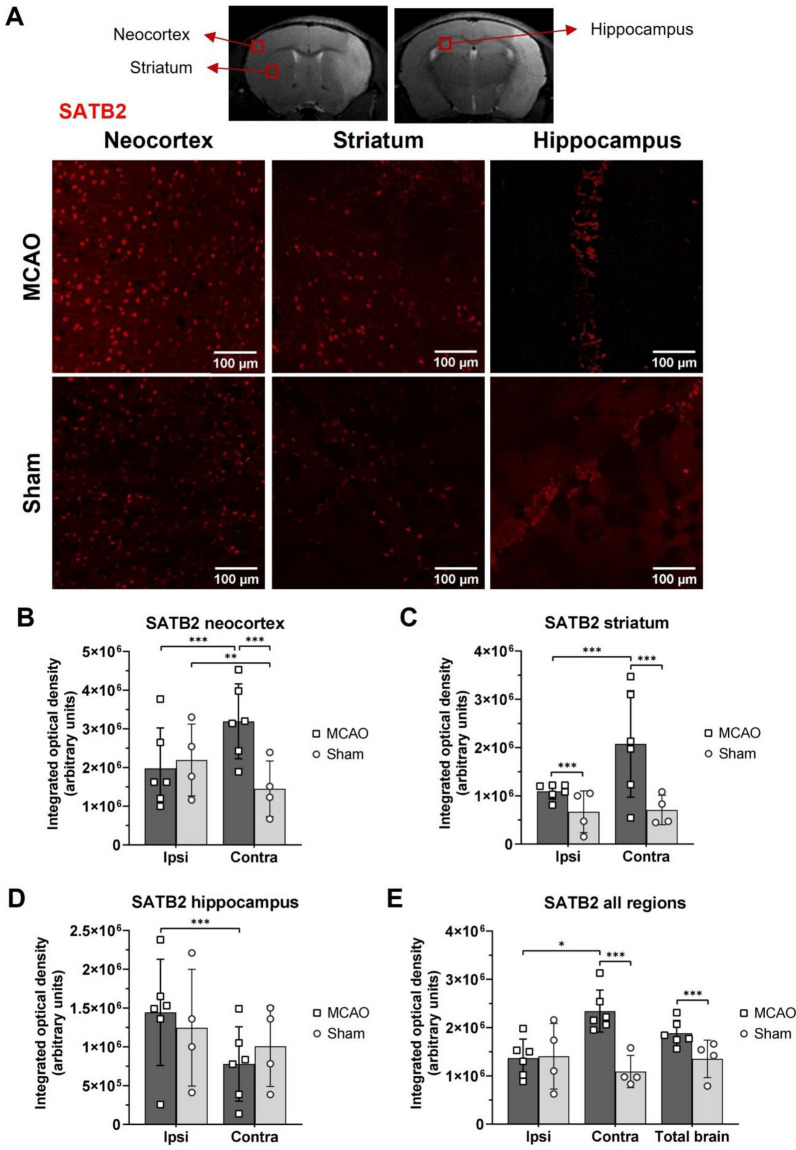
Immunohistochemistry results of SATB2 in the mouse brains isolated 7 days after MCAO or Sham surgery. (**A**) Representative immunofluorescent images of SATB2 (red) expression in the contralateral neocortex, striatum and hippocampus of MCAO and Sham animals. Regions of interest displayed on immunofluorescent images are marked with the red square on the representative MR images of the mouse brain. (**B–D**) Results of the integrated optical density measurements of SATB2 immunofluorescent signal in the neocortex (**B**), striatum (**C**) and hippocampus (**D**) of MCAO and Sham groups showed higher SATB2 in the contralateral neocortex and in the striatum. (**E**) Integrated optical density of SATB2 calculated for whole brain hemispheres and the entire brain of MCAO and Sham animals. The largest difference between the experimental groups was observed for contralateral brain hemisphere. The statistical significance was tested using nested ANOVA followed by Tukey’s multiple comparisons correction. Data are presented as mean ± standard deviation. *P < 0.050, **P < 0.010, ***P < 0.001.

Similarly, SATB2 expression was observed in the neocortex, striatum, and hippocampus of both brain hemispheres in MCAO and Sham experimental groups (Fig. 2). In the MCAO group higher integrated optical density of SATB2 than in Sham group was present in the ipsilateral striatum. However, in the contralateral hemisphere, as well as in contralateral neocortex and striatum, SATB2 presence was higher in the MCAO group than in the Sham group, as well as compared to the corresponding ipsilateral hemispheres (Fig. 2, Supplemental Table 2). The dominance of SATB2 involvement in the contralateral hemisphere response to ischemic injury was not present only in the hippocampus. As an unexpected result, the sham-operated animals showed an increase of SATB2 in the ipsilateral compared to the contralateral neocortex (*P* = 0.009) that probably resulted from the sham procedure or the anesthesia exposure during imaging sessions (Fig. 2). Although the sham operation is generally considered not to have any adverse effects, the findings from our own group show that the ligation of Common carotid artery during the sham procedure causes chronic hypoperfusion in the brain^25^.

Taken together, the results showed increased expression of both, BCL11B and SATB2 after ischemic lesion of mouse brain. Their expression after brain ischemia showed different spatial patterns. While the BCL11B was higher in both hemispheres but more pronounced in the ipsilateral hemisphere, SATB2 increased predominantly in the contralateral brain hemisphere in comparison to the Sham group.

**Figure 3:**
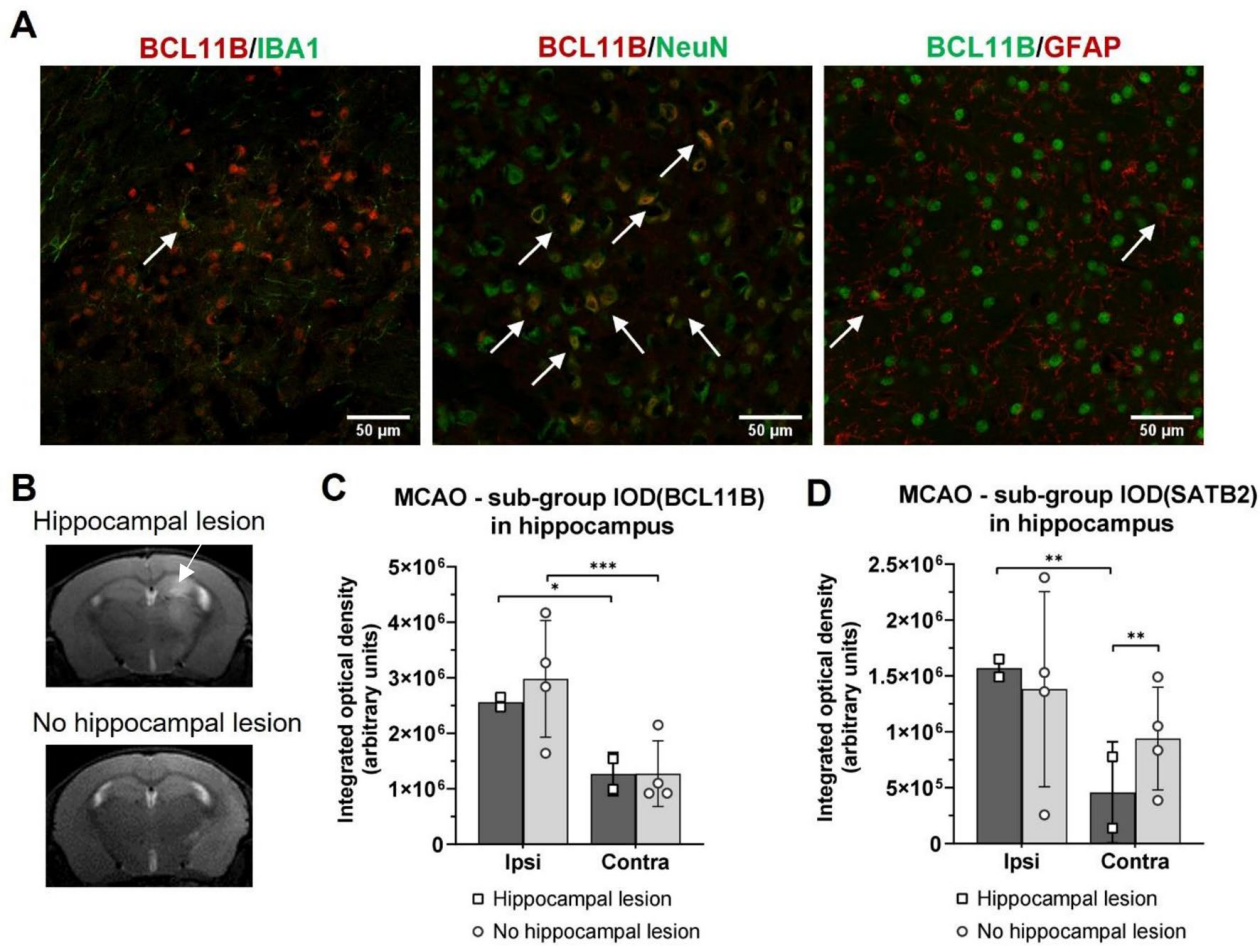
(**A**) Representative immunohistochemistry images of BCL11B (red or green) co-expression with microglial marker IBA1 (green), neuronal marker NeuN (green) and astrocytic marker GFAP (red). Co-expressions are marked with white arrows. Majority of BCL11B co-expressed with neuronal NeuN, although some co-expression with IBA1 and GFAP was also visible. (**B**) Representative T2-weighted MR images of mouse brain taken 7 days after tMCAO showing hippocampus affected by ischemic lesion (hyperintensive signal, arrow) and cortico-striatal lesion. (**C–D**) Results of integrated optical density measurements of BCL11B (**C**) and SATB2 (**D**) in hippocampus of the MCAO sub-groups. The animals were grouped based on the presence (n = 2) or absence of hippocampal lesion (n = 4). Hippocampal lesion decreased SATB2 expression on contralateral hemisphere compared to ipsilateral and to the sub-group without hippocampal lesion. The expression of BCL11B wasn’t affected by the presence of hippocampal lesion. The statistical significance was tested using nested ANOVA followed by Tukey’s multiple comparisons correction. Data are presented as mean ± standard deviation. *P < 0.050, **P < 0.010, ***P < 0.001.

To validate if the higher values of BCL11B in the MCAO group were from the reactivation of corticogenesis-related transcriptional factor in the neurons, double immunohistochemistry was made with neuronal and glial markers. BCL11B was selected for the co-expression analysis since its increase was prevalent in the ipsilateral hemisphere affected by experimental intervention, where the eventual repair processes of the lesion would occur. BCL11B had abundant overlap with the neuronal marker NeuN, while some minor co-expressions with microglial and astrocytic markers were observed (Fig. 3A). This as well excluded that the measured increased values of BCL11B resulted due some other type of cells migrating to the injured brain like the invading T-lymphocytes, known to express BCL11B.

Another validation was done due to the variable size of ischemic lesion optionally including the hippocampus^26^. In our study most of the MCAO operated animals (4/6) had ischemic lesion in the neocortex and striatum, but not in hippocampus, and in the remaining two animals the hippocampus was also affected (Fig. 3B). These differences could influence the optical density results for the hippocampal region. Therefore, we divided the MCAO animals in two sub-groups based on the presence of ischemic lesion in the hippocampus and tested the differences in the BCL11B and SATB2 expression between them. Presence of lesion in hippocampus did not influence the expression of BCL11B (Fig. 3C). At the other hand, the sub-group analysis of SATB2 indicated that hippocampal lesion decreased its expression in the contralateral brain hemisphere compared to the ipsilateral hemisphere (*P* = 0.002) and to the sub-group without hippocampal lesion (*P* = 0.003) (Fig. 3D). These results should be considered preliminary due to the small sample size. The decrease of SATB2 in the contralateral hippocampus affected by the lesion could be explained by the prominent neuron loss in the contralateral hippocampus caused by the ipsilateral ischemic damage^27^.

### Brain ischemia increased co-expression of BCLL11B and SATB2

Since integrated optical densities of both, BCL11B and SATB2 were higher following ischemic brain lesion, the next aim was to characterize their co-expression. For this purpose, the number of positive cells in the selected regions of interest was counted, including the number of BCL11B, SATB2, and their merged signals for BCL11B, SATB2, and the DAPI signal. The number of merged signals was normalized to the number of DAPI signal representing a total number of cells.

**Figure 4:**
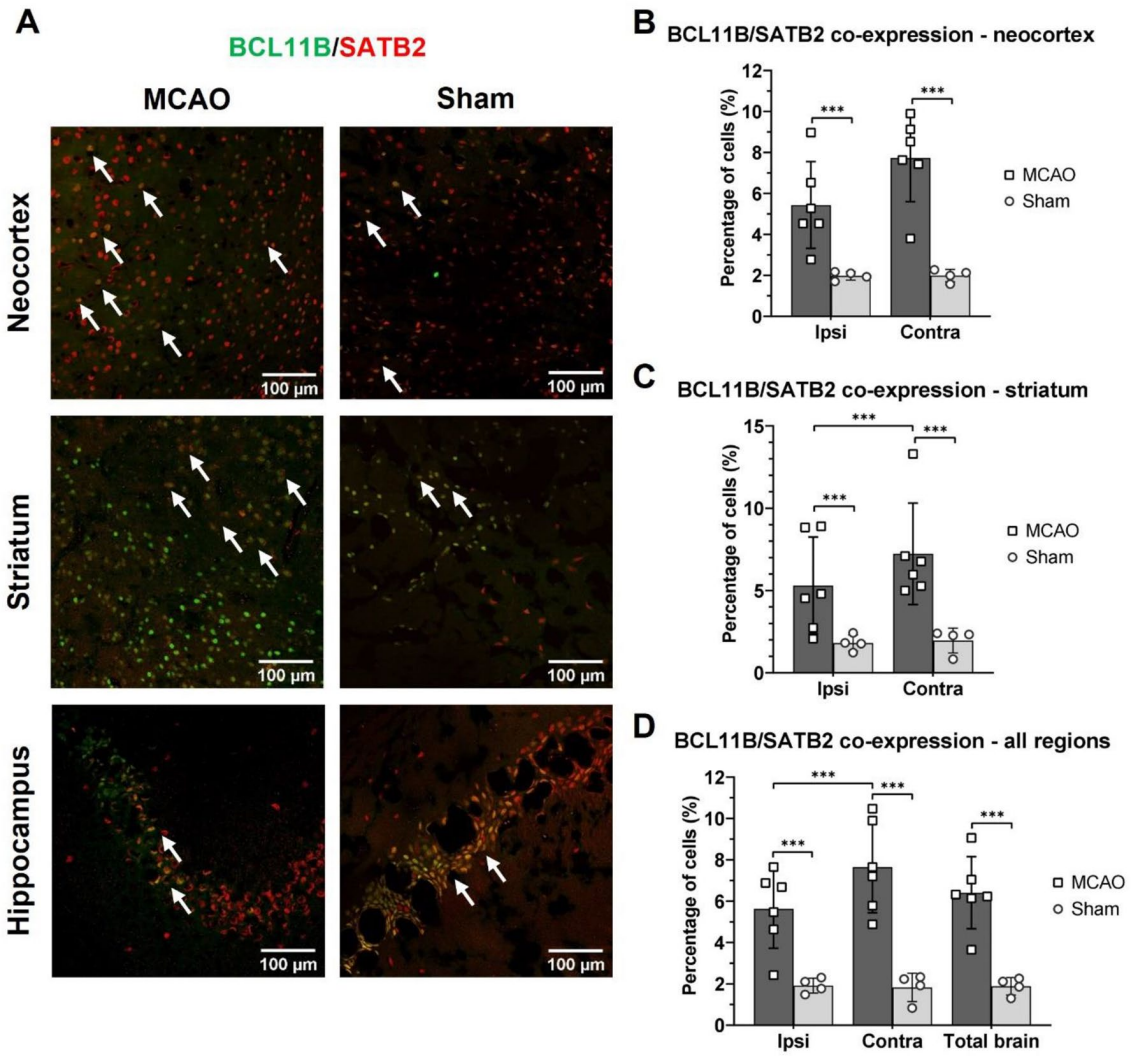
Immunohistochemistry results for the co-expression of BCL11B and SATB2 in the adult mouse brains isolated 7 days after MCAO or Sham surgery. (**A**) Representative immunohistochemistry images of the over-lapping (white arrows) BCL11B (green) and SATB2 (red) signals in the contralateral neocortex, striatum, and hippocampus of MCAO and Sham groups. Immunofluorescent background was removed while processing the images with the aim to emphasize co-expression and avoid false-positive results. Percentage of BCL11B-SATB2 co-expression (of the total number of cells in the region of interest) in the neocortex (**B**) and striatum (**C**) of MCAO and Sham animals. The increase in BCL11B and SATB2 co-expression after ischemia was present in the neocortex and striatum. (**D**) Percentage of BCL11B-SATB2 co-expression calculated for the brain hemispheres and the entire brain of MCAO and Sham animals. Brain ischemia increased co-expression of BCL11B and SATB2 in both brain hemispheres. The data was analyzed with nested ANOVA followed by Tukey’s correction for multiple comparisons. Data are presented as mean ± standard deviation. ***P < 0.001.

The co-expression of BCL11B and SATB2 fluorescent signals was observed in the neocortex, striatum and hippocampus, on both brain hemispheres, and in MCAO and Sham groups (Fig. 4A, Supplemental Table 3). However, only the results for neocortex and striatum could be quantified, because in the hippocampus the individual signals were dense and overlapping, making cell counting unreliable.

Quantification revealed higher percentage of BCL11B and SATB2 co-expression in the neocortex and striatum, in both contralateral and ipsilateral hemispheres and in the entire brain of MCAO animals than in their Sham counterparts (Fig. 4). Moreover, the co-expression was higher in the contralateral hemisphere, in particular in the contralateral striatum than in the ipsilateral hemisphere of the MCAO animals.

The increase of co-expression after brain ischemia was not only due to a general increase of expression of the each individual transcription factor, but it was a specific effect [25.27 ± 7.58% of BCL11B positive neurons co-expressed SATB2 vs. 8.37 ± 4.64% (*P* < 0.001) in the Sham group, and vice versa 15.92 ± 7.21% of SATB2 positive neurons at the same time expressed BCL11B vs. 4.31 ± 2.03% of the Sham group (*P* < 0.001)].

### BCL11B co-expressed with ATF3, marker of brain repair after ischemia, but not with the detrimental HDAC2

To investigate if BCL11B is co-expressed with other post-ischemic markers, it was tested with either beneficial ATF3 (activating transcription factor 3) or detrimental chromatin remodeler histone deacetylase HDAC2^28–32^. BCL11B was selected for the co-expression analysis since its increase was prevalent in the ipsilateral hemisphere affected by experimental intervention, where the eventual repair processes of the lesion would occur. Since all 3 markers are transcriptional regulators located in the nucleus, the overlap of their nuclear localization was counted and presented as a percentage of cells with the merged signal in relation to the total number of BCL11B positive cells.

**Figure 5:**
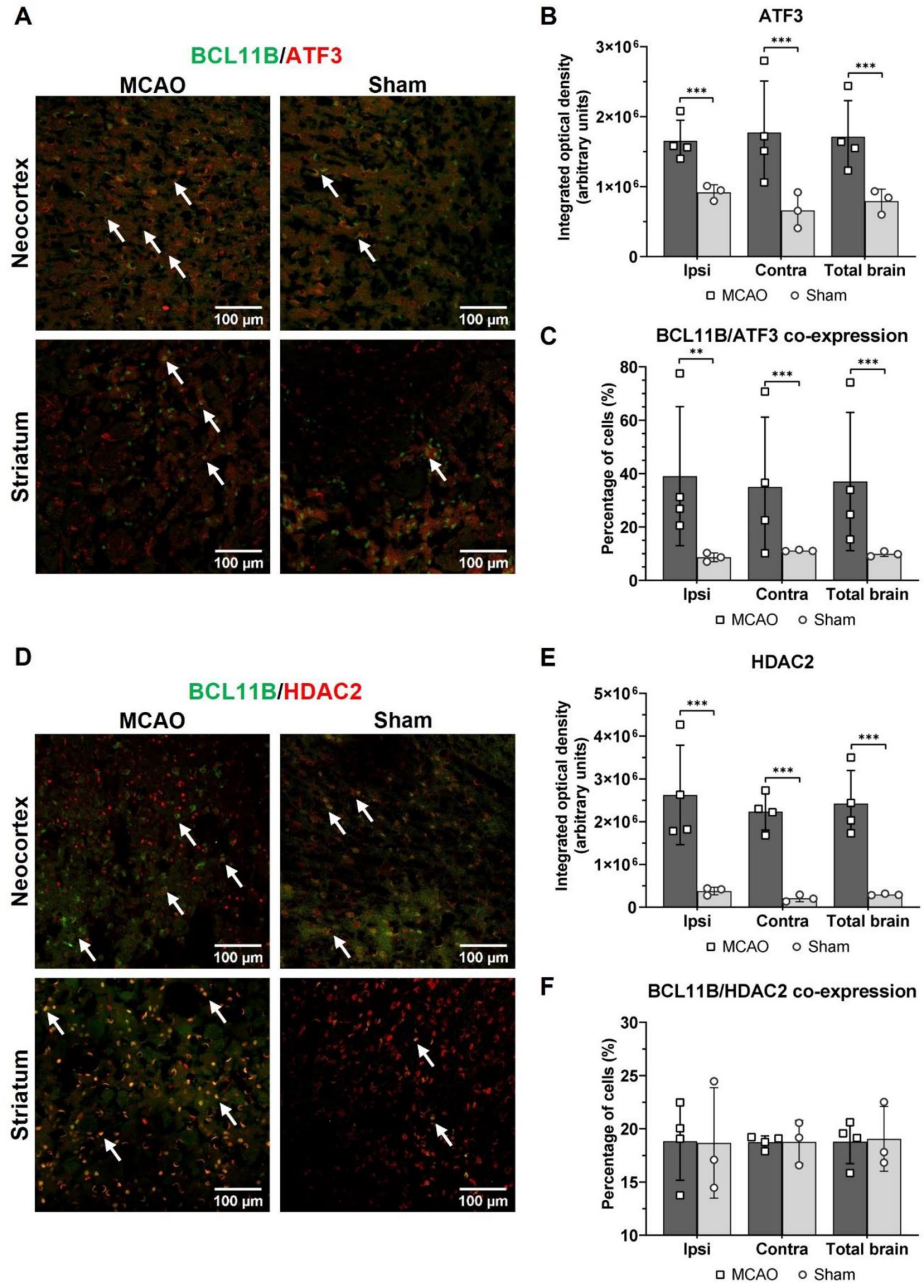
Immunohistochemistry results of ATF3 and HDAC2 expression in mouse brains isolated 7 days after MCAO or Sham surgery. (**A**) Representative immunohistochemistry images of BCL11B (green) and ATF3 (red) co-expression (white arrows) in the ipsilateral neocortex and striatum of MCAO and Sham groups. (**B**) Integrated optical density of ATF3 in the ipsilateral, contralateral and entire brain of MCAO and Sham animals. (**C**) Percentage of BCL11B and ATF3 co-expression in the ipsilateral, contralateral and entire brain of MCAO and Sham animals. (**D**) Representative immunohistochemistry images of BCL11B (green) and HDAC2 (red) co-expression (noted with white arrows) in the ipsilateral neocortex and striatum of MCAO and Sham experimental groups. (**E**) Integrated optical density of HDAC2 in ipsilateral, contralateral and entire brain of MCAO and Sham animals. (**F**) Percentage of BCL11B and HDAC2 co-expression in ipsilateral, contralateral and entire brain of MCAO and Sham animals. The co-expression was represented as a percentage of the total number of BCL11B positive cells. Background signal on immunofluorescence images was removed while processing images to emphasize the co-expression. Statistical significance for the presented results was tested by nested ANOVA followed by Tukey’s correction for multiple comparisons. Data are presented as mean ± standard deviation. **P < 0.010, ***P < 0.001.

Before quantifying the merged signal, integrated optical density for ATF3 and HDAC2 was measured to confirm their over-expression after brain ischemia. As expected, the increased integrated optical density of ATF3 and HDAC2 was observed in MCAO group, compared to their Sham counterparts (Fig. 5).

The co-expression of BCL11B and ATF3 was observed in all analyzed regions in both MCAO and Sham groups (Fig. 5, Supplemental Table 4). The higher percentage of co-localized BCL11B and ATF3 in MCAO group was present in the ipsilateral hemisphere (*P* = 0.006) and in the entire brain (*P* < 0.001), but not specifically in the contralateral hemisphere when compared to the Sham group. To contrast the observed increase of co-expression with beneficial ATF3, we also quantified the co-expression of BCL11B with the detrimental HDAC2 (Fig. 5, Supplemental Table 5). The results showed comparable co-expression of BCL11B and HDAC2 in MCAO and Sham groups, further supporting the beneficial effect of BCL11B expression. However, it should be noted that in both groups the share of co-expression of these two markers was relatively high (less than 20%).

**Figure 6:**
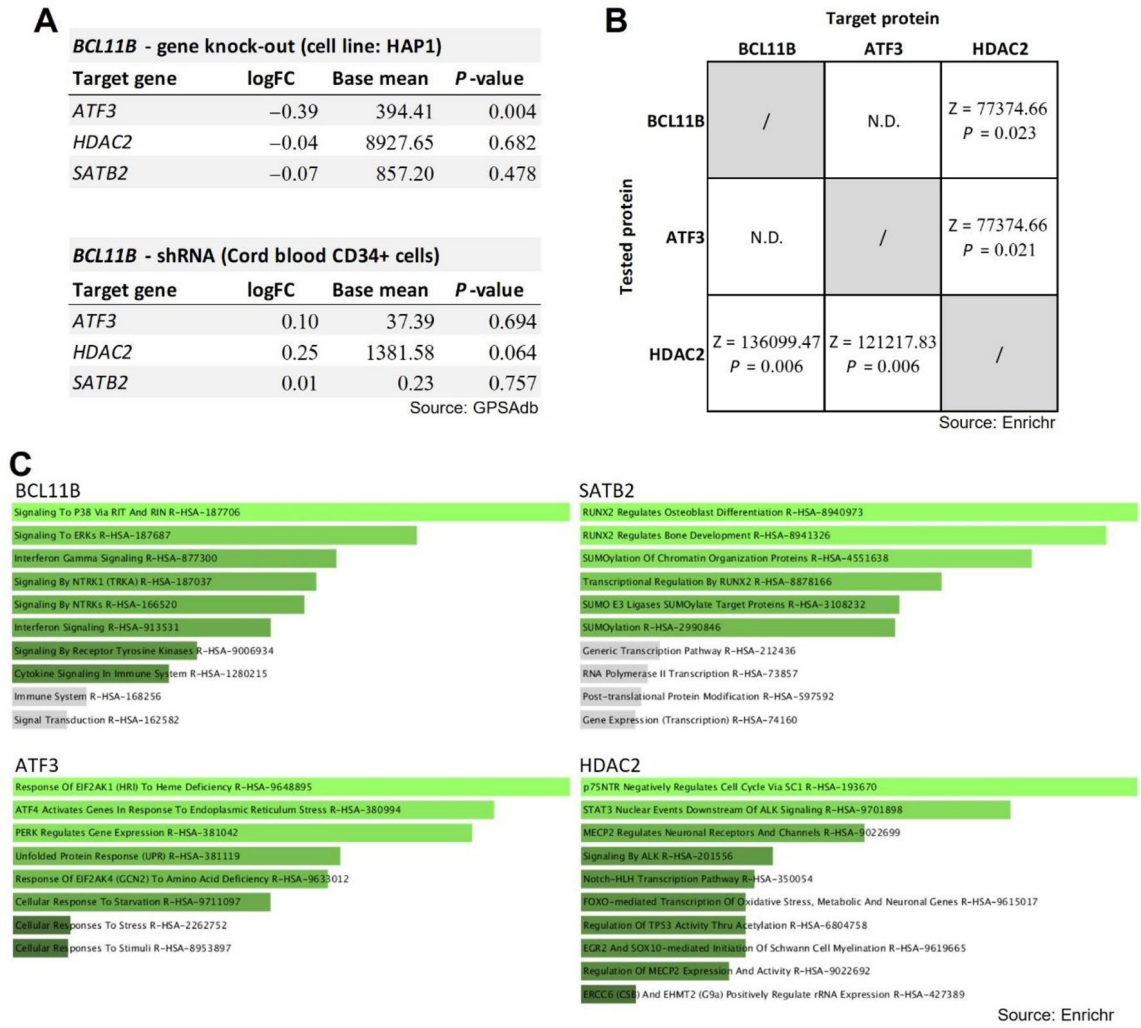
Results of in silico gene perturbation analysis. (**A**) Gene perturbations after *Bcl11b* knock-out or knock-down with shRNA. Cell line HAP1 was available as a model of *Bcl11b* gene knock-out, while shRNA-mediated gene knock-down was performed on cord blood CD34 + cells. Positive logFC indicates increased expression while negative values suggest decrease in targeted gen’s expression after *Bcl11b* knock-out/down. *Atf3* was significantly decreased in the *Bcl11b* knock-out model. Gene perturbations after *Satb2* gene silencing were not tested as they were not available in the GPSAdb database. (**B**) Results of potential protein–protein interactions analysed in the database Transcription Factor PPIs using *Enrichr* tool. For every identified interaction *Z*-score and *P*-value are noted. Interactions between BCL11B and ATF3 were not identified, however HDAC2 had potential interactions with both BCL11B and ATF3. N.D. (not determined) represents the protein–protein interactions that were not available in the database. Protein–protein interactions for SATB2 were not available in the database and therefore not shown. (**C**) Results of the pathway analysis in the Reactom 2022 database (*Enrichr* tool) show all tested proteins had their own distinct signalling pathways. The images show top 10 over-represented pathways for the proteins of interest: BCL11B, SATB2, ATF3 and HDAC2. Green colour indicates the statistical significance of over-representation (*P* < 0.050) with smaller *P*-values shaded in lighter colour. Over-representation of pathways shaded in grey colour was not statistically significant (*P* > 0.050).

To address the implications of increased BCL11B and AFT3 co-expression after ischemic stroke, an in silico gene perturbation analysis was performed using genetic perturbation similarity analysis (GPSA). GPSAdb database search provided for in silico analysis two different types of *Bcl11b* gene deficiency: *Bcl11b* gene knock-out in HAP1 cell line, and knock-down with shRNA in cord blood CD34 + cells. In a *Bcl11b* knock-out model the expression of *Atf3* gene was significantly down-regulated (logFC = − 0.39, *P* = 0.004), while no differences in the *Hdac2* and *Satb2* expression were present (Fig. 6A). The limitation of in silico approach was that CNS cells weren’t utilized as a model, possibly obscuring the results.

In the next step we analysed the potential protein–protein interactions between BCL11B, ATF3 and HDAC2. The database search did not find potential protein–protein interactions between BCL11B and AFT3, however HDAC2 had potential interactions with both BCL11B and ATF3 (Fig. 6B). Over-represented pathways of BCL11B, SATB2, ATF3 and HDAC2 were compiled, however similarities in signalling pathways were not revealed, highlighting their different functions (Fig. 6C).

To sum up, gene perturbation analysis revealed BCL11B and AFT3 had potentially distinct cellular functions, however BCL11B might be involved in regulation of *Atf3* gene transcription which coincided with their increased co-expression after the ischemic stroke.

### Increased expression of BCL11B and SATB2 after brain ischemia correlated with the functional recovery

**Figure 7:**
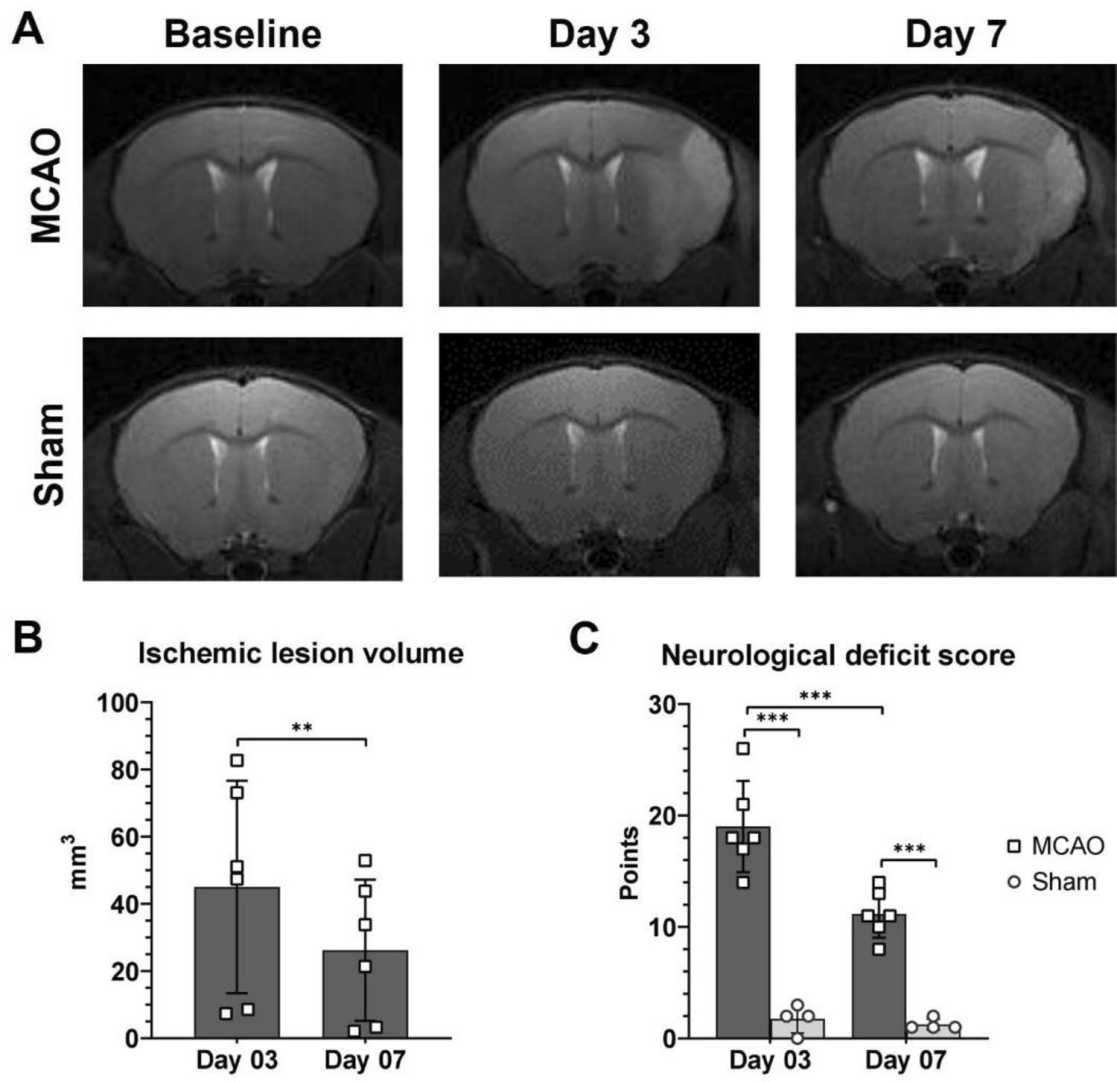
(**A**) Representative T2-weighted MR images of MCAO and Sham operated animals. Hiperintensive signal on the left hemisphere of the MCAO group represented the ischemic brain lesion. Ischemic lesion was not present after sham operations. The Sham group was imaged at same time points as MCAO group to eliminate the effect of imaging procedure and anesthesia on the results. (**B**) Temporal changes of the lesion volumes in MCAO group. The lesion volume significantly decreased by day 7, as measured with t-test for paired samples. (**C**) Changes in the neurological deficit score for MCAO and Sham animals. MCAO group had higher neurological deficit than Sham group, and it improved till day 7. The statistical differences were calculated using two-way ANOVA for repeated measurements followed by Sidak’s and Tukey’s corrections for multiple comparisons. Data are presented as mean ± standard deviation. *P < 0.050, **P < 0.010, ***P < 0.001.

To monitor the progression of ischemic brain lesion of every individual animal, they underwent MRI before, and 3 and 7 days after tMCAO or Sham operation (Fig. 7A). Volume of ischemic brain lesion was calculated by manually delineating hyperintensive signal on T2 maps. The volumetric analysis of the brain lesion in MCAO group revealed the largest lesion 3 days after tMCAO (45.02 ± 31.61 mm^3^), and subsequent reduction of its size on day 7 (26.19 ± 21.01 mm^3^, *P* = 0.005) (Fig. 7B). The longitudinal imaging allowed for comparing the size of ischemic lesion on days 3 and 7 and the lesion reduction rate was calculated by expressing the lesion volume on day 7 as a percentage of its volume measured on day 3. The overall lesion reduction rate in the experiment was 50.70 ± 15.82%.

The functional consequences of the ischemic lesion were estimated by neurological deficit score. Before the induction of ischemic brain lesion all animals had a perfect score (0 points), without measurable neurological deficits. After the induction of ischemic lesion neurological deficit score in the MCAO group followed the temporal dynamics of lesion size. The greatest deficit was present 3 days after tMCAO (19.00 ± 4.10 points) followed by a significant reduction on day 7 (11.17 ± 2.14 points, *P* < 0.001) (Fig. 7C). Similarly, the functional recovery rate was defined as the neurological deficit score on day 7 calculated as a percentage of neurological deficit score on day 3. The average recovery rate was 59.04 ± 4.18%. Despite lacking the ischemic lesion, Sham animals presented with the minimal neurological deficits after the surgery (Day 3: 1.75 ± 1.26 points; Day 7: 1.25 ± 0.50 points), however this functional deficit was significantly smaller compared to MCAO experimental group (*P* < 0.001) (Fig. 7C).

**Figure 8:**
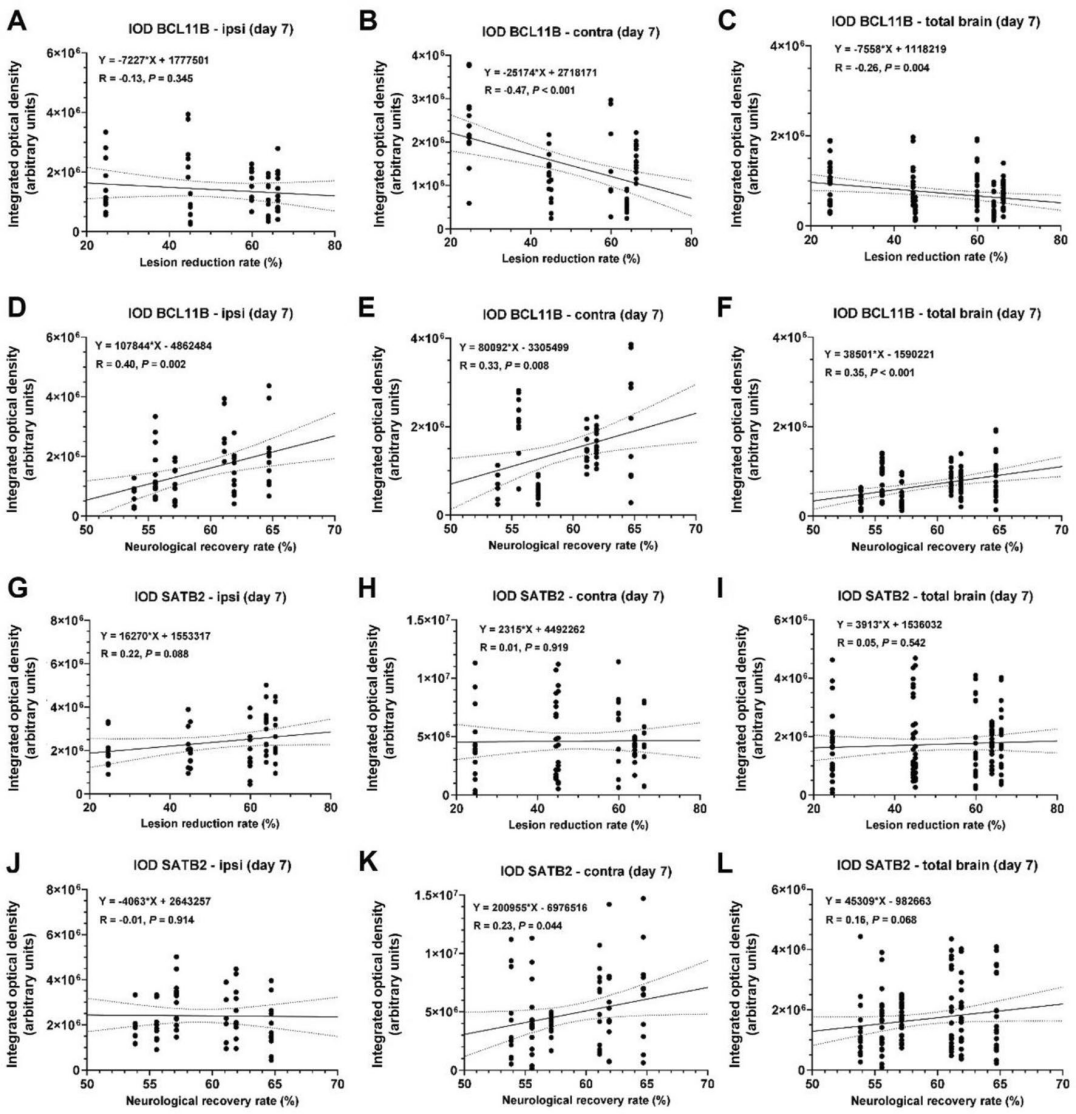
Correlations of BCL11B (**A–F**) and SATB2 (**G–L**) expression (measured by integrated optical density) with the lesion reduction rate and neurological recovery rate in MCAO group. Full line represents linear regression line, while dotted curves represent 95% confidence interval. For every correlation equation of linear regression line, Pearson’s correlation coefficient and the statistical significance of the correlation are reported.

**Figure 9:**
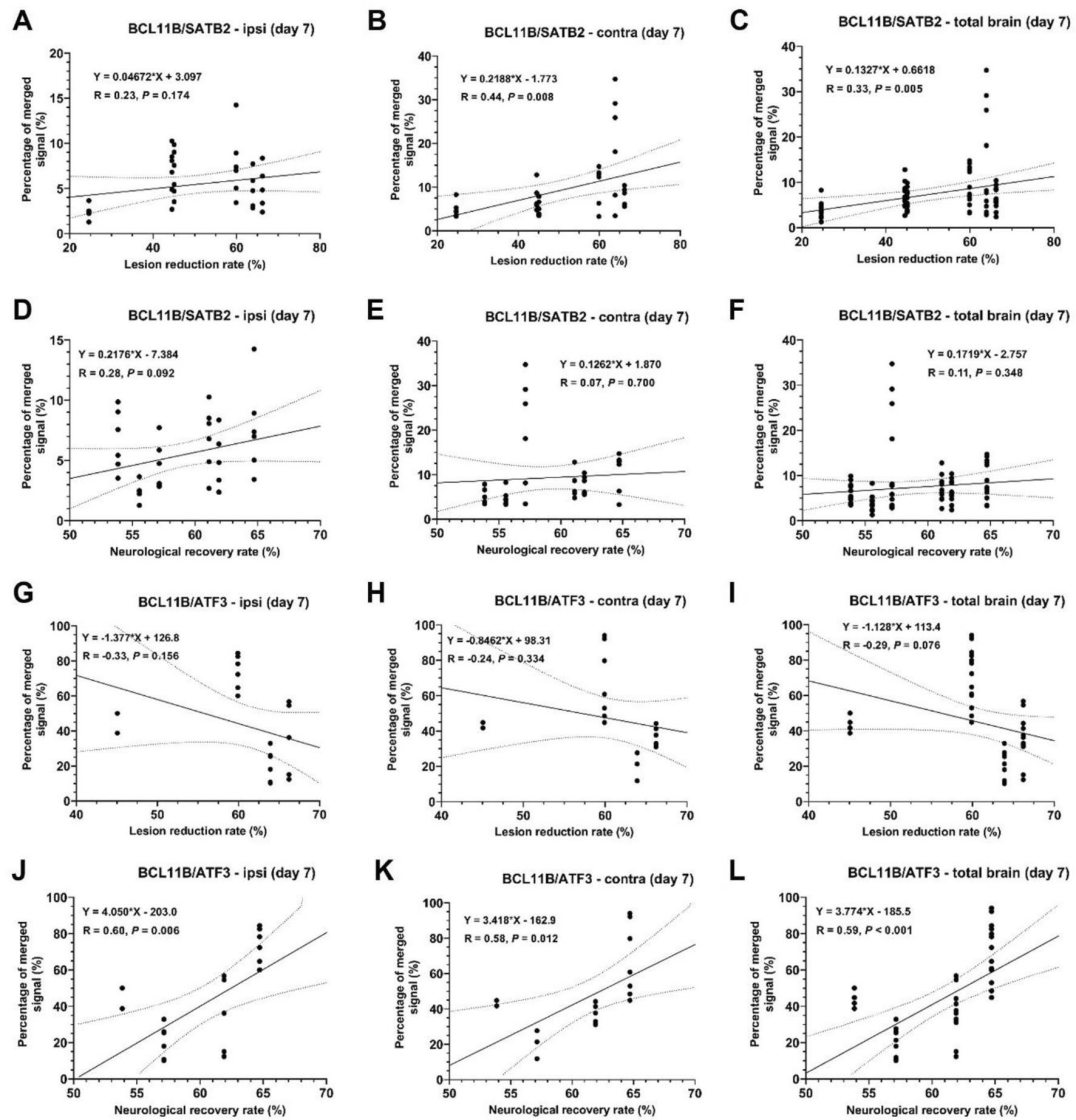
Correlations of BCL11B-SATB2 (**A–F**) and BCL11B-ATF3 (**G–L**) co-expression (presented as percentage of total cell) with the lesion reduction rate and neurological recovery rate in MCAO group. Full line represents linear regression line, while dotted curves represent 95% confidence interval. For every correlation equation of linear regression line, Pearson’s correlation coefficient and the statistical significance of the correlation are reported.

In order to link BCL11B and SATB2 expression to the repair after ischemic lesion, the immunohistochemistry results of MCAO group were correlated with the lesion reduction rate and functional recovery rate. BCL11B expression had positive correlation with the functional recovery rate, but not with the lesion reduction rate (Fig. 8A–F). There was even negative correlation with lesion reduction rate in the contralateral hemisphere (Fig. 8B,C). Similarly, SATB2, which was in previous experiments shown to predominantly increase in the contralateral hemisphere, indeed in the contralateral hemisphere correlated with the functional recovery rate, but there was no correlation with lesion reduction rate (Fig. 8G–L). Moreover, the degree of BCL11B and SATB2 co-expression had positive correlation with both lesion reduction rate and functional recovery rate (Fig. 9A–F). Additionally, the percentage of BCL11B and ATF3 co-expressing cells also had positive correlation with the functional recovery rate suggesting their combined beneficial effect on functional recovery (Fig. 9G–L). These results indicated the potential beneficial role of both BCL11B and SATB2 activation on the post-stroke functional recovery.

To contrast the correlations with recovery rates, the immunohistochemistry results were also correlated with damaging processes, including the volume of ischemic lesion and neurological deficit score on days 3 and 7. BCL11B expression on ipsilateral and contralateral hemisphere had negative correlation with lesion volume on days 3 and 7 (Supplemental Fig. 1). SATB2 expression on contralateral hemisphere showed negative correlation with neurological deficit score (Supplemental Fig. 2). Furthermore, BCL11B and SATB2 co-expression on contralateral hemisphere correlated positively with lesion volume, but at the same time negative correlation with neurological deficit score was observed (Supplemental Fig. 3). Co-expression of BCL11B and ATF3 did not correlate with the ischemic damage (Supplemental Fig. 4). Taken together, an increase of BCL11B and SATB2 after ischemia was accompanied with their positive correlations with the functional recovery and negative correlations with ischemic damage, suggesting their beneficial role in brain ischemia.

## Discussion

This study confirmed the initial hypothesis that the expression of corticogenesis-related transcription factors BCL11B and SATB2 can be reactivated after brain ischemic lesion in the adult mouse. Moreover, we demonstrated a correlation of this increase with functional recovery. We could not test direct involvement of both BCL11B and SATB2 in the recovery after stroke, as respective mutant mice do not survive after birth and show a plethora of defects, including brain malformations^33,34^. Therefore, before the eventual preclinical intervention in their brain regulation, we carried out a correlative investigation and combined the expression study with the progression of ischemic lesion in the individual animals monitored by in vivo MRI and neurological deficit scoring. This allowed to correlate the expression of BCL11B or SATB2 to the structural and functional outcomes, indicating their potential involvement into post-stroke events.

To elucidate if BCL11B and SATB2 could be involved in the recovery after brain ischemic damage, we investigated two paradigms. In the first set of experiments, we demonstrated an increased co-expression of BCL11B with the beneficial ATF3, rather than with detrimental HDAC2, after ischemic lesion. These results were corroborated further in the second set of experiments where we correlated expression levels results with lesion reduction and neurological recovery rates. Positive correlation of BCL11B and ATF3 co-expression with neurological recovery rate confirmed that the BCL11B/AFT3 co-expression is a relevant marker of positive functional outcomes. Furthermore, BCL11B and SATB2 expression positively correlated with neurological recovery rate suggesting their beneficial role in damage repair after ischemia. One of the possible explanation for their beneficial effect in brain ischemia is the ability to recruit components of NuRD chromatin remodeling complex, specifically HDAC1, since their activation is associated with the development of smaller ischemic lesions, improved neurological outcome and attenuated neuronal death^4,6,35,36^. Moreover, BCL11B expression had negative correlation with lesion volume, while SATB2 expression showed no correlation with detrimental factors, supporting their beneficial role in recovery after brain ischemia. Likewise, the increase of BCL11B and SATB2 co-expression after ischemia correlated with the lesion reduction and neurological recovery rates suggesting their combined effect on functional recovery.

It is well established that functional recovery after stroke is facilitated by axonal sprouting in the peri-infarct area and in the corresponding cortical regions of the contralateral hemisphere resulting in the compensation of the lost synapsis^37–39^. We suggest neurons co-expressing BCL11B and SATB2 might be involved in axonal outgrowth processes after brain ischemia since such neurons in the developing neocortex project to corpus callosum and into brain stem nuclei^8^. This assumption could also explain why the observed co-expression increased in both hemispheres after ischemia.

Although the transcriptional regulators BCL11B and SATB2 are commonly associated with the corticogenesis, they are also constitutively expressed in the adult mouse brain^10,11^. BCL11B and SATB2 are rarely co-expressed in neurons and distinct spatial patterns are observed in the cortical layers as well as in the sub-cortical structures, mirroring the different functions of BCL11B and SATB2 positive neurons^2,4,8^. The differences in spatial pattern and subsequent functional diversities are achieved by SATB2 recruitment on *Bcl11b* locus, and subsequent inhibition of *Bcl11b* gene transcription^4^. However, the BCL11B and SATB2 co-expression in neurons is enabled by LMO4 binding on *Bcl11b* locus that prevents SATB2 recruitment, and in healthy brain only a small amount of neurons expresses both transcriptional modulators^4,8^. Their co-expression results in distinct function of double-positive neurons, diverse from the neurons positive solely for BCL11B or SATB2^4,8^. This complementarity of their function was also reflected in the BCL11B and SATB2 distribution after brain ischemia. In tMCAO model utilized in this study, the ischemic damage affects only one brain hemisphere, while the pattern of the BCL11B and SATB2 over-expression increased in both hemispheres as well in the lesion adjacent regions (neocortex, striatum and hippocampus). BCL11B increase was observed over both brain hemispheres, however SATB2 increase after brain ischemia was predominant in the contralateral hemisphere that was not directly affected by ischemic damage. Higher SATB2 expression in the contralateral brain hemisphere enabled BCL11B activation in the ipsilateral hemisphere due to decreased inhibition of *Bcl11b* gene promoter. Similarly, since the expressions of both BCL11B and SATB2 were increased in the contralateral hemisphere after ischemia, this is also where we identified the highest frequency of their co-expression. Our finding of elevated co-expression suggested that the levels of LMO4 might be increased after ischemia, as well. Furthermore, we might conclude that these double-positive neurons had different role in post-stroke recovery compared to neurons that express only one of these transcriptional modulators.

In attempt to clarify the functional consequences of BCL11B over-expression after ischemia we quantified the co-expression with previously proven markers of beneficial and damaging processes after brain ischemia. BCL11B was selected for the co-expression analysis since its increase was dominant in the ipsilateral affected hemisphere where the potential repair processes of the lesion would occur. ATF3 is a transcriptional regulator with leucine zipper domain, and it activates in response to a variety of cellular stress signals, including the ischemia^40^. The mice with inhibited ATF3 develop larger ischemic lesions accompanied by more severe neurological deficits and prominent post-stroke inflammation^28,29^. Additionally, the increase of ATF3 after brain ischemia is associated with reduction of neuronal death and more efficient synaptic recovery^29,41,42^. On the other hand, HDAC2 is epigenetic factor that removes acetyl from histones thus making chromatin less accessible for transcriptional factors^43^. After brain ischemia, the increase of HDAC2 levels coincides with the neuronal death, larger ischemic lesions and impaired functional recovery^30–32^. Despite the substantial over-expression of both ATF3 and HDAC2 after brain ischemia, only the increase in ATF3 and BCL11B co-expression was observed, while BCL11B and HDAC2 co-expression was not altered, indicating increase of BCL11B was associated with the beneficial processes after brain ischemia. Furthermore, positive correlation of BCL11B and ATF3 co-expression with neurological recovery rate supported these findings. In silico gene perturbation study identified BCL11B as a possible regulator of *Atf3* gene transcription, although protein–protein interactions and similarities in the signaling pathways were not identified. Therefore, the results suggested that BCL11B and ATF3 might have synergistic effect promoting functional recovery after stroke.

The preclinical stroke research is rarely concerned with the dynamic of the events, but when time is taken in account, various treatments show correlation with functional recovery after ischemic brain lesion^22,44–46^. Having the advantage of in vivo imaging we tried to quantify two measures of recovery after ischemia, one based on volumetric data of lesion, and other based on simultaneously determined neurological deficit scores. These were defined as the lesion reduction and neurological recovery rates, and they were calculated on day 7 as a change compared to day 3 after ischemia. These time points were specifically chosen since both neurological deficit score and volume of ischemic lesion reach peak 3 days after ischemia onset, and the majority of damage rapidly reduces until day 7^22,46^.

The limitation of this study is that, although we have provided the temporal pattern of the progression of the ischemic lesion, the temporal dynamics of BCL11B and SATB2 activity remained unknown. Since their activity correlated with the better recovery rate, we suggest they might be active mainly during chronic phase of ischemic stroke, when the majority of functional recovery occurs^47^. However, further research is needed to confirm this hypothesis. Moreover, the expression study could not make a conclusion about the function of the tested gene or the directionality or causality of the relationship between the genes. To further extend on implications of BCL11B and SATB2 on functional recovery after ischemia, dynamics of axonal sprouting processes should be studied in detail. Advances in generalized deep learning network for fractional anisotropy reconstructions could be relevant for in vivo measurements of white matter damage in ischemic lesion, as well as sprouting mediated by the markers of corticogenesis.

In conclusion, corticogenesis-related transcriptional modulators BCL11B and SATB2 increased in the sub-acute phase of the ischemic lesion of adult mouse brain. Their potential involvement into recovery after brain ischemia was claimed on the finding that they are co-expressed with beneficial ATF3. Moreover, the expression level of SATB2 and BCL11B correlated with functional recovery. BCL11B and SATB2 appear as potential molecular markers for predicting better outcomes of the stroke recovery as well as potential targets for therapeutical interventions after ischemic stroke.

## Materials and methods

### Ethics statement

All animal handling and surgery was approved by the Ethics Committee of the University of Zagreb, School of Medicine (approval number 380–59-10106-17-100/100) and by the Committee of the Ministry of Agriculture Republic of Croatia (approval number UP/I-322-01/17-01/45). The experimental design followed ARRIVE guidelines for animal research^48^. All procedures conformed to the Ethical Codex of the Croatian Society for Laboratory Animal Science and to the EU Directive 2010/63/EU on the protection of animals used for scientific purposes.

### Experimental groups and study design

The study was carried out on 12–16 weeks old male mice C57BL6-Tyr^c-Brd^/J bred at the animal facility of the Croatian Institute for Brain Research. All animals were housed in the same environment; in a single housing room with temperature (22 ± 2 °C) and humidity control, under 12/12 h light/dark cycles. Water and food were available ad libitum.

A total of 12 animals was included in the experiments and the mice were randomly divided into 2 experimental groups: sham operated animals (n = 4) as a control group (depicted as Sham in text), and animals that underwent tMCAO (transient Middle Cerebral Artery occlusion) (n = 8) as a treatment group (depicted as MCAO in text). Two animals from MCAO group (2/8, 25%) did not survive until the study end, and were not included in the analysis, therefore the MCAO group consisted of 6 animals.

Both experimental groups underwent longitudinal MR imaging of the brain to confirm the absence of ischemic lesion in Sham group, and to monitor the progression of ischemic lesion in MCAO group. Imaging data were complemented with the neurological deficit test. Seven days after the surgery, all animals were sacrificed, and brains were isolated for the purpose of immunohistochemistry. Expression of corticogenesis markers BCL11B and SATB2 was compared between the experimental groups along with the additional transcriptional factors connected with the beneficial (ATF3) and detrimental (HDAC2) role in brain ischemia. Finally, the results of immunohistochemistry for the MCAO group were correlated with the stroke outcome.

### Transient middle cerebral artery occlusion (tMCAO)

Induction of unilateral ischemic lesion of mouse brain was performed by Koizumi method of transient occlusion of the left middle cerebral artery (MCA) as described previously^49^. Briefly, mice were anaesthetized with 4% isoflurane (Isoflurane, Abbott, UK) inhalation anesthesia, which was kept around 2% for anesthesia maintenance. A silicon rubber-coated 6–0 monofilament (Doccol Corporation, Sharon, MA USA) was introduced in the left middle cerebral artery via common carotid artery for 1 h, followed by 7 days reperfusion. Sham operated animals were subjected to the anesthesia and filament introduction/retrieval without an occlusion period. Mice were kept on a heated surface for the next 24 h. Following surgery, all animals received intraperitoneal injection of analgesic buprenorphine (dosage 0.05 mg/kg) once a day for 3 days to achieve post-surgical pain relief, and intraperitoneal injection of 30% glucose (dissolved in saline solution, dosage 250 μL) to prevent rapid weight loss and dehydration. Animas were weighted before the surgery and weight was monitored on all time points.

### Magnetic resonance imaging (MRI)

All animals underwent MR imaging of the brain prior to the surgery, 3 and 7 days after the procedure. Sham operated animals were also imaged in all time points to eliminate the potential impact of imaging procedure and anesthesia exposure on the expression of transcriptional modulators in the brain. Magnetic resonance imaging was performed on a 7 T system (BioSpec 70/20 USR with Paravision 6.0.1. software version, Bruker Biospin, Germany) in a Tx/Rx configuration, using an 86 mm transmit volume coil (MT0381, Bruker Biospin, Germany) for transmitting (Tx) and a 2-element mouse brain surface receive coil (MT0042, Bruker Biospin, Germany) for receiving (Rx). Prior to imaging, animals were anesthetized in a heated induction chamber with a mixture of 70/30% N_2_/O_2_ containing 4% isoflurane (Isoflurane, Abbott, UK) and during imaging inhalation anesthesia was maintained with 1–1.5% isoflurane in a 70/30% N_2_/O_2_ mixture. Animals were placed in a supine position inside a water-heated Bruker mouse bed (Bruker, Germany) and the position of their head was secured with the help of tooth and ear bars. The respiration rate was monitored during the whole scan and kept in the range 80–100 breath/min by adjusting the anesthesia. Body temperature was monitored by rectal temperature probe (Medres, Cologne, Germany) and was maintained at 37 ± 0.5 °C by controlling the temperature of water-heated bed and the additional cover with a feedback-controlled circulating heating pump.

The scan protocol consisted of a preparatory phase and main scans. In the preparatory phase, first a low-resolution GE pilot scan (Multi-Slice Localizer) was performed, followed by an additional TurboRARE scan in the sagittal plane. The main scans included a high-resolution T2-weighted anatomical Turbo Spin-Echo sequence scan (TurboRARE, TE/TR = 33/3000 ms, matrix size 160 × 100, total time = 4 min) and a T2-map scan Multi-echo Spin-Echo sequence protocol (MSME, TE/TR = 7.5/3150 ms, matrix size 128 × 80, total time = 12 min). T2 maps were calculated from measured data using Paravision 6.0.1. software (Bruker Biospin, Germany) built-in post-processing macros. The field of view was set to 16 × 10 mm, slice thickness was 0.5 mm with a 0.1 mm gap between the slices, and the number of slices was 25.

The size of ischemic lesion and the size of contralateral and ipsilateral brain hemispheres were determined using manual delineation method in ImageJ (NIH, Bethesda, USA). Ischemic lesion was delineated on T2-map scans while brain hemispheres were delineated on T2-weighted scans.

### Neurological deficit scoring

Neurological deficit scoring test was performed before the surgery, 3 and 7 days after the procedure. Neurological score was assessed on a scale of 0–56, where 0 is a perfect score without any neurological impairment^22^. Animals were evaluated in 13 categories: appearance (hair, eyes, ears, posture), spontaneous activity, epileptic behavior, body symmetry, gait disturbances, climbing on a surface held at 45°, circling behavior, front limb symmetry, compulsory circling, and whisker response to light touch.

### Tissue collection

Seven days after the surgery animals were anesthetized by an intraperitoneal injection of 2.5% Avertin (Sigma–Aldrich, Germany) and then transcardially perfused with phosphate-buffered saline (PBS; 1X, pH 7.4) followed by 4% paraformaldehyde (PFA). Isolated brains were incubated overnight in 4% PFA, and gradually equilibrated in 30% sucrose solution (dissolved in PBS) before embedding into cryopreservation medium Tissue-Tek (O.C.T. compound, Sakura, USA). Samples were frozen and stored on − 20 °C. Brains were cut into 16 μm thick coronal sections with a Cryostat (Leica, Wetzlar, Germany). Two brain slices (distance from bregma: 0.5 mm and − 1.6 mm) were collected.

### Immunohistochemistry

Brain sections were vacuumed for 2 h on room temperature. Slides were then blocked for 1 h in 10% goat serum and 0.25% Triton X-100 (Sigma–Aldrich, Germany) dissolved in PBS. Primary antibodies dissolved in PBS containing 1% goat serum and 0.1% Triton X-100 were added and incubated overnight on the room temperature (Table 1). Afterwards, sections were incubated for 2 h at room temperature with corresponding secondary antibodies dissolved in PBS (Table 1). Finally, brain sections were incubated in DAPI solution (5 nM, Abcam, USA), covered with Fluorescence Mounting Medium S3023 (Dako, Denmark) and stored at + 4 °C.

**Table 1:** List and characteristics of primary and secondary antibodies used for immunohistochemistry.

Antibody	Host	Dilution	Type	Company
BCL11B	Rat	1:250	Primary	Abcam
SATB2	Rabbit	1:200	Primary	Abcam
ATF3	Rabbit	1:200	Primary	Sigma–Aldrich
HDAC2	Rabbit	1:250	Primary	Abcam
GFAP	Chicken	1:500	Primary	Abcam
IBA1	Rabbit	1:250	Primary	Wako
NeuN	Rabbit	1:400	Primary	Abcam
Alexa-Flour® 488	Goat anti Rat	1:500	Secondary	Invitrogen
Alexa-Flour® 546	Goat anti Rabbit	1:500	Secondary	Invitrogen
Alexa-Flour® 568	Goat anti Rat	1:500	Secondary	Abcam
Alexa-Flour® 633	Goat anti Rabbit	1:500	Secondary	Invitrogen
Alexa-Flour® 647	Goat anti Chicken	1:500	Secondary	Invitrogen

Slides were imaged on confocal microscope Olympus Fluorview 3000 (Olympus, Japan) equipped with 4 excitation lasers: 405 nm, 488 nm, 561 nm and 640 nm. Images were acquired using 20 × objective lens (UPlanSApo, Olympus, Japan) and resolution was set to 1024 × 1024 pixels. All images were taken using the same exposure parameters.

### Quantification of the immunofluorescent signal

For the quantification of immunofluorescent signal, one section was acquired on confocal microscope from neocortex, striatum and hippocampus surrounding the necrotic ischemic core, along with corresponding regions on the contralateral brain hemisphere. Immunofluorescence was quantified with the ImageJ (NIH, Bethesda, USA) software by measuring integrated optical density and by counting cells positive for specific markers^50^. Integrated optical density was utilized as a measurement of the expression for selected proteins. Prior to optical density measurement, the background was removed. For neocortex and striatum, integrated optical density was measured in 5 regions of interest, 0.40 × 0.40 mm each, and for hippocampus two regions of interest were selected. Two independent researchers manually counted immunofluorescent signals within 3 regions of interest (0.40 × 0.40 mm each) in neocortex and striatum on both brain hemispheres. Since all of the observed proteins are transcriptional factors located in the nuclei, the number of immunofluorescent signals corresponded to the number of cells positive for the specific signal. Signal/cell counting was performed for the specific markers, co-expression of two markers and DAPI. Before counting the merged signal, images were processed by completely removing the background with the aim of avoiding false-positive results. Number of DAPI signal corresponded to the total number of cells in the selected region of interest. Cell counting was not performed in the hippocampus region due to abundant over-lapping of the signal.

### In silico gene perturbation analysis

Gene perturbation study was carried out using genetic perturbation similarity analysis (GPSA) method on dataset provided by the open-source online tool GPSAdb^51^. Two different methods of genetic perturbations of *Bcl11b* gene were identified: gene knock-out and conditional knock-down by shRNA, and the analysis of down-stream genes affected by perturbation was done separately for both groups. To quantify the changes in gene expression that resulted from *Bcl11b* gene perturbation, the logarithmic value of fold change (log_2_FC) was collected indicating increased (positive values) or decreased (negative values) expression for the downstream genes of interest. Genetic perturbations for *Satb2* gene were not analysed since they were at this time not available in GPSAdb. Additionally, to complement the results of gene perturbation analysis, potential protein–protein interactions were assessed using over-representation analysis (ORA) method in the database Transcription Factor PPIs available at the open-access web based software Enrichr^52,53^. The protein–protein interactions for SATB2 were not available in the database and therefore could not have been analysed. The statistical significance of enrichment was calculated by comparing the selected gene set with the results of randomly chosen gene set, while Z-score and *P*-value were obtained using Fischer’s exact test with Benjamini–Hochberg correction for multiple hypotheses testing. The analysis of over-represented signalling pathways was performed using database Reactome 2022 on Enrichr platform and top ten over-represented pathways were extracted.

### Statistical analysis

Normality of distribution was tested using Shapiro–Wilk test and all stroke outcome data had normal distribution. Volume of ischemic lesion was analyzed by t-test for paired samples. Neurological deficit score was assessed by two-way ANOVA for repeated measurements followed by Tukey’s (for the differences between the experimental groups) and Sidak’s (for the differences between time points) multiple comparison tests.

Immunohistochemistry results failed the normality test, therefore they were transformed using natural logarithm function and the nested ANOVA was performed on the transformed values. Results for multiple regions of interest were collected for the single animal, therefore nested design ANOVA followed by Tukey’s multiple comparison test was applied. Linear regression between the stroke outcome in MCAO experimental group and immunofluorescence results was performed and Pearson’s correlation coefficient was calculated. All statistical analysis was performed in SPSS (version 25.0, IBM Corp., USA). Differences were considered statistically significant with *P* < 0.050. All data are presented as mean ± SD.

## Supplementary Information

Supplementary Information.

## Data Availability

The datasets used and/or analyzed during the current study are available from the corresponding author on reasonable request.
