# Supplementary material for: Reactivation of corticogenesis-related transcriptional factors BCL11B and SATB2 after ischemic lesion of the adult mouse brain
Source: Sci Rep. 2023 May 26;13:8539. doi: 10.1038/s41598-023-35515-8 (PMC10220074; doi:10.1038/s41598-023-35515-8)
Supplement: Supplementary file 1 — Supplementary Information. [file 41598_2023_35515_MOESM1_ESM.docx]

**Reactivation of corticogenesis-related transcriptional factors BCL11B and SATB2 after ischemic lesion of the adult mouse brain**

Sanja Srakočić; Dunja Gorup; Dominik Kutlić; Ante Petrović; Victor Tarabykin; Srećko Gajović

# **SUPPLEMENTAL RESULTS**

**Supplemental Table 1:** Immunohistochemistry results of BCL11B in the mouse brains isolated 7 days after MCAO or Sham surgery. Integrated optical density (in arbitrary units) was measured in neocortex, striatum and hippocampus in the regions surrounding ischemic lesion and in the corresponding regions on the contralateral brain hemisphere. The results are represented as average ± SD.

|  | **MCAO** | | **Sham** | |
| --- | --- | --- | --- | --- |
|  | **Ipsi** | **Contra** | **Ipsi** | **Contra** |
| **Neocortex** | 1.43×10^6^ ± 5.92×10^5^ | 1.48×10^6^ ± 7.28×10^5^ | 5.00×10^5^ ± 1.66×10^5^ | 4.85×10^5^ ± 1.09×10^5^ |
| **Striatum** | 1.71×10^6^ ± 1.36×10^5^ | 1.81×10^6^ ± 1.20×10^6^ | 4.43×10^5^ ± 3.02×10^5^ | 4.69×10^5^ ± 2.11×10^5^ |
| **Hippocampus** | 2.84×10^6^ ± 8.43×10^5^ | 1.27×10^6^ ± 4.90×10^5^ | 7.84×10^5^ ± 3.86×10^5^ | 9.67×10^5^ ± 6.33×10^5^ |
| **Combined** | 1.80×10^6^ ± 9.72×10^5^ | 1.62×10^6^ ± 8.94×10^5^ | 5.50×10^5^ ± 1.92×10^5^ | 7.44×10^5^ ± 2.27×10^5^ |
| **Total brain** | 1.70×10^6^ ± 7.52×10^5^ | | 5.86×10^5^ ± 1.19×10^5^ | |

**Supplemental Table 2:** Immunohistochemistry results of SATB2 in the mouse brains isolated 7 days after MCAO or Sham surgery. Integrated optical density (in arbitrary units) was measured in neocortex, striatum and hippocampus in the regions surrounding ischemic lesion and in the corresponding regions on the contralateral brain hemisphere. The results are represented as average ± SD.

|  | **MCAO** | | **Sham** | |
| --- | --- | --- | --- | --- |
|  | **Ipsi** | **Contra** | **Ipsi** | **Contra** |
| **Neocortex** | 2.10×10^6^ ± 1.32×10^6^ | 3.07×10^6^ ± 7.93×10^5^ | 2.19×10^6^ ± 9.29×10^5^ | 1.45×10^6^ ± 7.18×10^5^ |
| **Striatum** | 1.09×10^6^ ± 1.61×10^5^ | 2.08×10^6^ ± 1.10×10^6^ | 6.68×10^5^ ± 4.33×10^5^ | 7.07×10^5^ ± 3.05×10^5^ |
| **Hippocampus** | 1.44×10^6^ ± 6.83×10^5^ | 7.79×10^5^ ± 4.79×10^5^ | 1.25×10^6^ ± 7.53×10^5^ | 1.01×10^6^ ± 5.16×10^5^ |
| **Combined** | 1.37×10^6^ ± 3.93×10^5^ | 2.34×10^6^ ± 4.34×10^5^ | 1.41×10^6^ ± 6.77×10^5^ | 1.09×10^6^ ± 3.33×10^5^ |
| **Total brain** | 1.88×10^6^ ± 2.66×10^5^ | | 1.35×10^6^ ± 3.89×10^5^ | |

**Supplemental table 3:** Immunohistochemistry results for the co-localization of BCL11B and SATB2 in the adult mouse brains isolated 7 days after MCAO or Sham surgery. Percentage of BCL11B and SATB2 co-expression was evaluated in neocortex and striatum in the regions surrounding ischemic lesion and in the corresponding regions on the contralateral brain hemisphere. The number of over-lapping signal was normalized to the number of DAPI signal, representing a total number of cells. The results are represented as average ± SD.

|  | **MCAO** | | **Sham** | |
| --- | --- | --- | --- | --- |
|  | **Ipsi** | **Contra** | **Ipsi** | **Contra** |
| **Neocortex** | 5.43 ± 2.12% | 7.74 ± 2.14% | 1.98 ± 0.21% | 1.99 ± 0.30% |
| **Striatum** | 5.31 ± 2.94% | 7.23 ± 3.08% | 1.81 ± 0.49% | 1.96 ± 0.75% |
| **Combined** | 5.62 ± 1.90% | 7.65 ± 2.21% | 1.91 ± 0.36% | 1.83 ± 0.69% |
| **Total brain** | 6.41 ± 1.74% | | 1.88 ± 0.42% | |

**Supplemental Table 4:** Immunohistochemistry results for the co-localization of BCL11B and ATF3 in the adult mouse brains isolated 7 days after MCAO or Sham surgery. Percentage of BCL11B and ATF3 co-expression was evaluated in the hemisphere surrounding ischemic lesion and in the corresponding regions on the contralateral brain hemisphere. The number of over-lapping signal was normalized to the number of DAPI signal, representing a total number of cells. The results are represented as average ± SD.

|  | **MCAO** | **Sham** |
| --- | --- | --- |
| **Ipsi** | 39.05 ± 26.06% | 8.65 ± 1.64% |
| **Contra** | 35.00 ± 26.17% | 11.16 ± 0.26% |
| **Total brain** | 37.02 ± 25.89% | 9.90 ± 0.93% |

**Supplemental Table 5:** Immunohistochemistry results for the co-localization of BCL11B and HDAC2 in the adult mouse brains isolated 7 days after MCAO or Sham surgery. Percentage of BCL11B and HDAC2 co-expression was evaluated in the hemisphere surrounding ischemic lesion and in the corresponding regions on the contralateral brain hemisphere. The number of over-lapping signal was normalized to the number of DAPI signal, representing a total number of cells. The results are represented as average ± SD.

|  | **MCAO** | **Sham** |
| --- | --- | --- |
| **Ipsi** | 18.84 ± 3.68% | 18.67 ± 5.19% |
| **Contra** | 18.74 ± 0.59% | 18.77 ± 2.02% |
| **Total brain** | 18. 79 ± 2.06% | 19.05 ± 3.04% |

## **Additional correlations results**


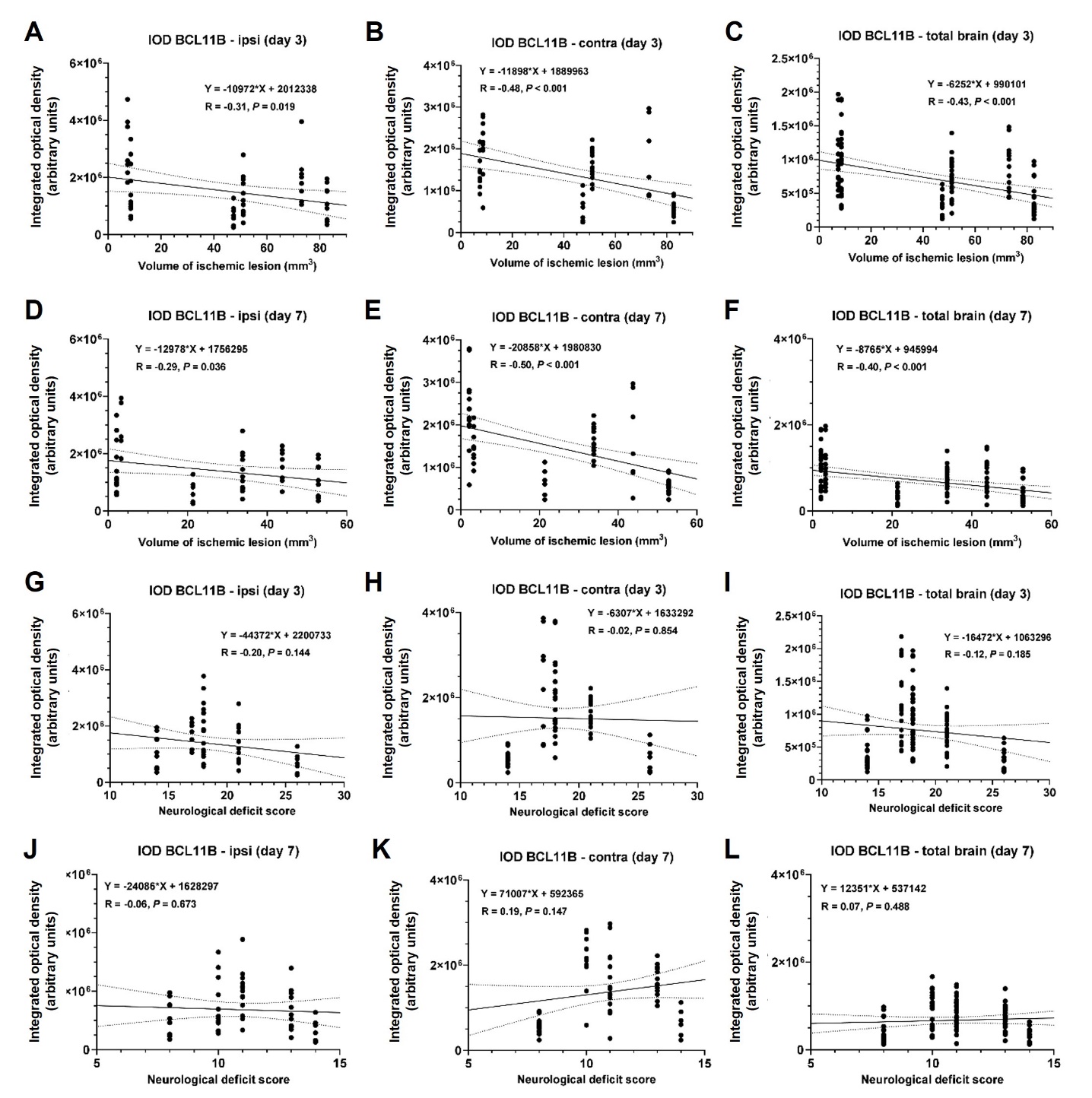


**Supplemental Figure 1:** Correlation of BCL11B expression (measured by integrated optical density) with volume of ischemic lesion and neurological deficit 3 and 7 days after ischemia in MCAO group. Full line represents linear regression line, while dotted curves represent 95% confidence interval. For every correlation equation of linear regression line, Pearson’s correlation coefficient and the statistical significance of the correlation are reported.


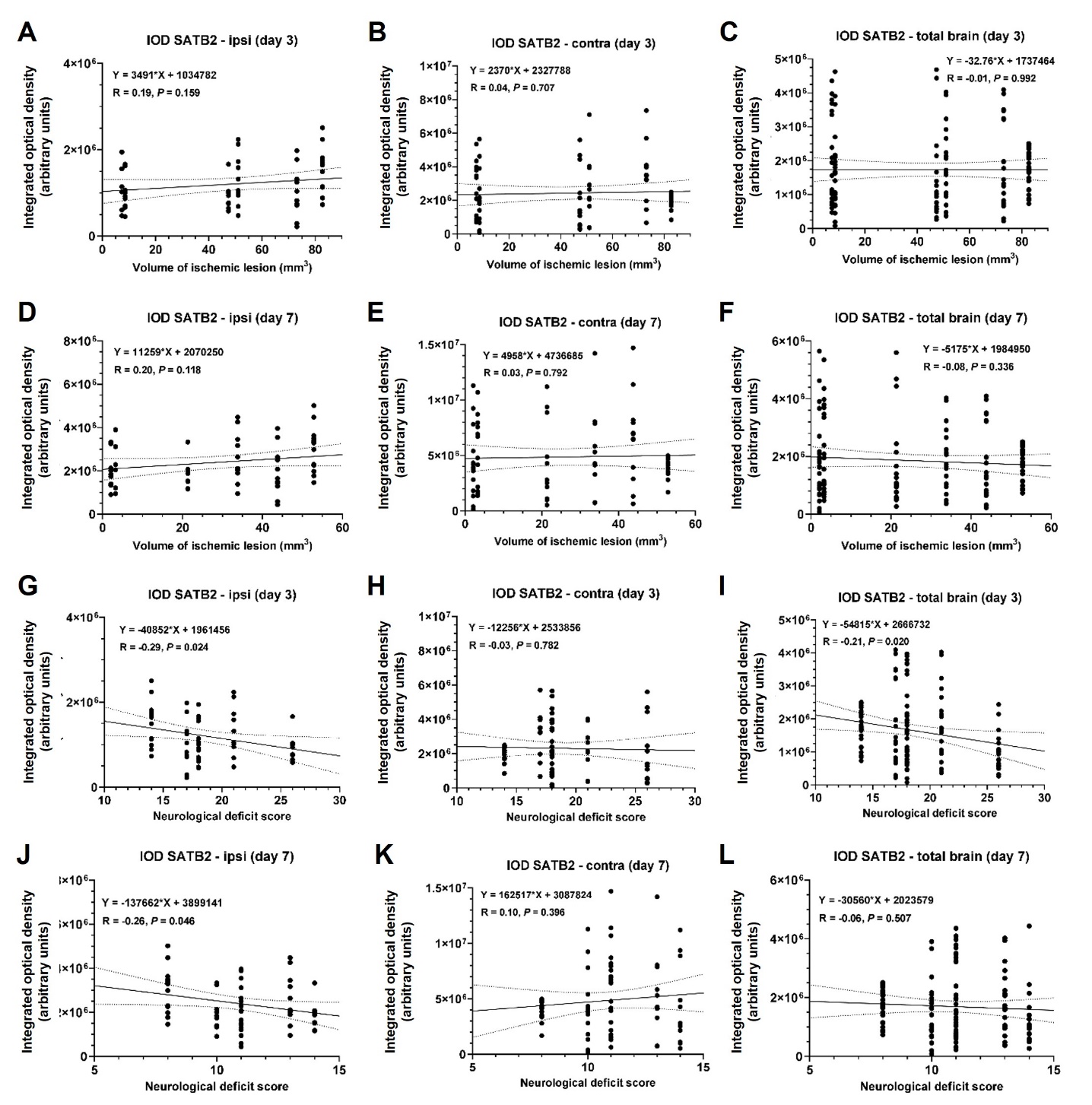


**Supplemental Figure 2:** Correlation of SATB2 expression (measured by integrated optical density) with volume of ischemic lesion and neurological deficit 3 and 7 days after ischemia in MCAO group. Full line represents linear regression line, while dotted curves represent 95% confidence interval. For every correlation equation of linear regression line, Pearson’s correlation coefficient and the statistical significance of the correlation are reported.


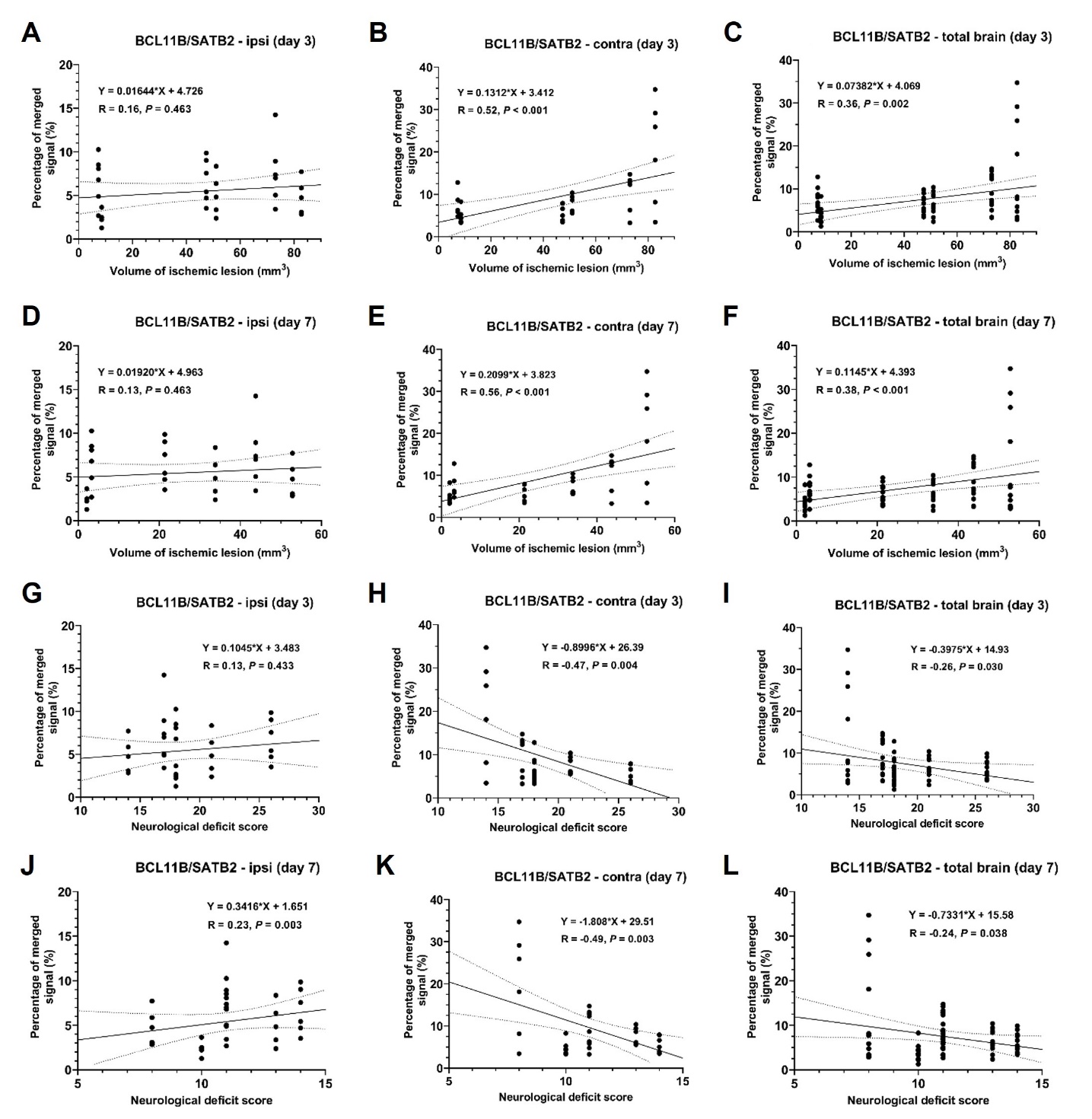


**Supplemental Figure 3:** Correlations of BCL11B-SATB2 co-expression (presented as percentage of total cell) with volume of ischemic lesion and neurological deficit 3 and 7 days after ischemia in MCAO group. Full line represents linear regression line, while dotted curves represent 95% confidence interval. For every correlation equation of linear regression line, Pearson’s correlation coefficient and the statistical significance of the correlation are reported.


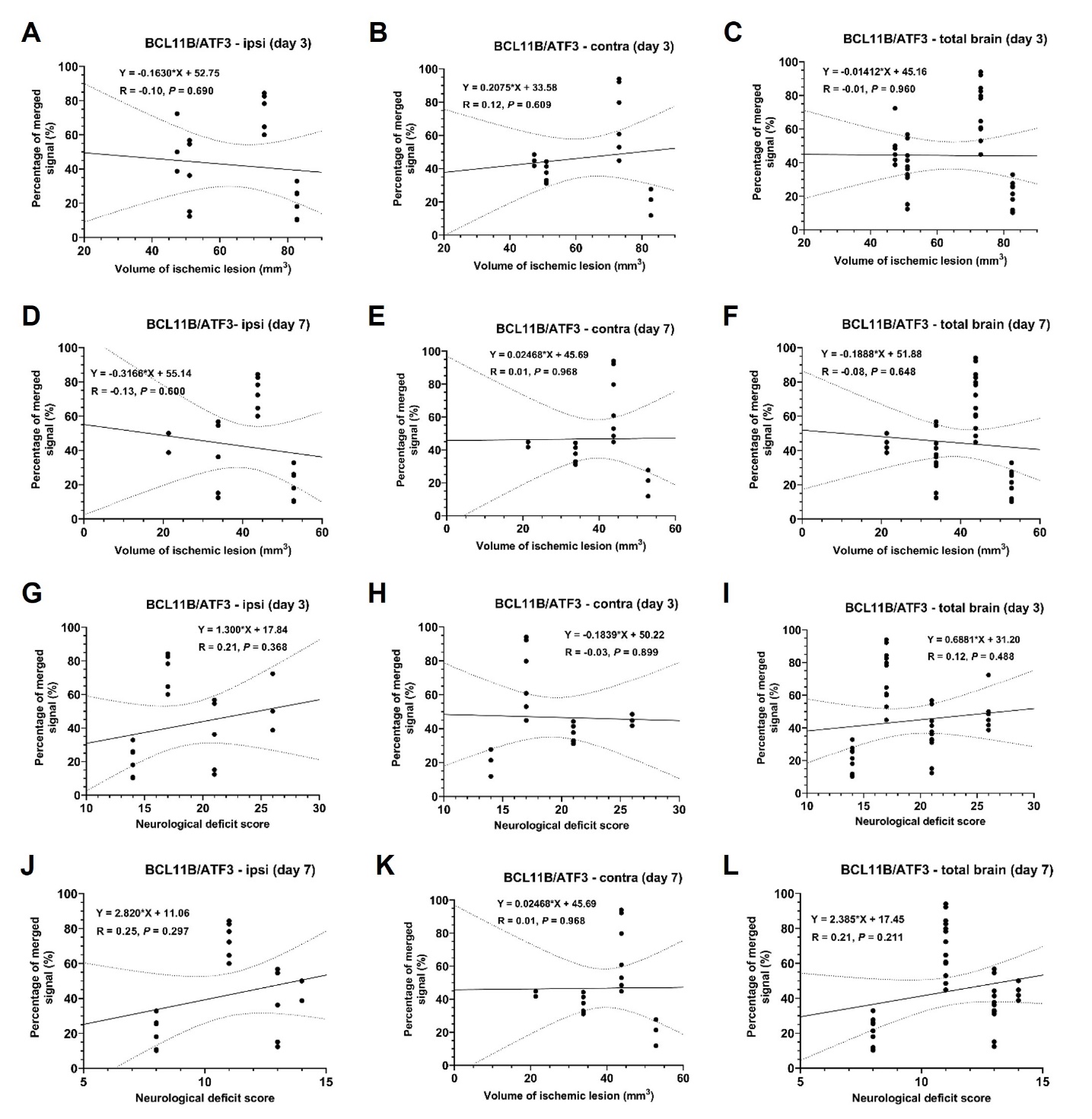


**Supplemental Figure 4:** Correlations of BCL11B-ATF3 co-expression (presented as percentage of total cell) with volume of ischemic lesion and neurological deficit 3 and 7 days after ischemia in MCAO group. Full line represents linear regression line, while dotted curves represent 95% confidence interval. For every correlation equation of linear regression line, Pearson’s correlation coefficient and the statistical significance of the correlation are reported.
